# Role of multimodal approach to curing anxiety disorders

**DOI:** 10.1192/j.eurpsy.2021.1335

**Published:** 2021-08-13

**Authors:** O. Kudinova, B. Mykhaylov, T. Chorna

**Affiliations:** 1 Psychotherapy, Kharkiv Medical Academy of Postgraduate Education, Kharkiv, Ukraine; 2 Psychiatry, National Academy of Postgraduate Education, Kiev, Ukraine; 3 Psychiatry, Vinnysya National Pirogov Memorial Medical University, Vinnysya, Ukraine

**Keywords:** multimodal approach, episodic paroxismal disorders, anxiety disorders

## Abstract

**Introduction:**

On the basis of complex clinical anamnestic, clinical psychopatological, pathopsychological research, data were obtained about reasons and conditions of formation, abnormal clinical psychopathological structure, syndrome peculiarities of emotional disfunctions for patients with episodic paroxismal disorders, generalized anxiety disorders and mixed anxiously depressed disorders.To realize the aim and tasks of the research, 145 patients were examined with anxiety disorders, that passed the stationary course of treatment.

**Objectives:**

The purpose of the research was to discover emotional disturbance peculiarities for anxiety disorder patients with different origins of pathological syndromes.

**Methods:**

The basic method was a group psychotherapy with the elements of rational, positive, suggestive and family psychotherapy. In relation to disfunctions of emotional sphere, cognitive-behavioral therapy (CBT) was used for the phobic-depressive and anxious-depressed disorders.

**Results:**

Decrease in general level of anxiety and internal anxiety was obtained for most patients. No spontaneous emergence of fear was practically observed. While active interviewing, patients stated that their former worries and fears have lost actuality and apparent emotional colouring, somatic-vegetative correlates of anxious states disappeared. Up to the end of the therapy course, a sense of calmness was attained as a base-line for the background emotional state. Considerable reduction of symptomatic of the depressed circle also took place. Patients’ mood increased, their interests broadened, patients started to feel joy and optimism.

**Conclusions:**

To correct emotional disfunction of patients with episodic paroxismal disorders, generalized anxiety disorders and mixed anxiously depressed disorders, psychotherapeutic correction system is optimal to use, which is built based on stepwise and multimodal principles.

